# Diagnosing Sporadic Creutzfeldt-Jakob Disease in a Patient with a Suspected Status Epilepticus in the Intensive Care Unit

**DOI:** 10.1155/2013/630141

**Published:** 2013-04-04

**Authors:** Harm J. van der Horn, Peter H. Egbers, Michaël A. Kuiper, Wouter J. Schuiling

**Affiliations:** ^1^University Medical Centre Groningen, GN, The Netherlands; ^2^Department of Intensive Care, The Medical Centre Leeuwarden, FR Leeuwarden, The Netherlands; ^3^Department of Neurology, The Medical Centre Leeuwarden, FR Leeuwarden, The Netherlands

## Abstract

*Objective*. Several tests are available in the diagnostics of sporadic Creutzfeldt-Jakob disease (sCJD); however, none of these is conclusive. We review the values of these tests, from an intensive care unit (ICU) perspective. *Methods*. Case report and review of the literature. *Results*. A 53-year-old woman initially presenting with psychiatric symptoms developed myoclonus and was admitted 1 month later to the ICU with a suspected nonconvulsive status epilepticus and respiratory insufficiency, probably due to extensive antiepileptic drug therapy. Typical MRI and EEG findings and a positive 14-3-3 protein led to the diagnosis of sCJD. All treatments were terminated, and autopsy confirmed sCJD. *Conclusions*. Clinical signs combined with MRI, EEG, and 14-3-3 and/or tau protein determination might be sufficient to diagnose or exclude sCJD and may therefore prevent the application of unnecessary diagnostic tests.

## 1. Introduction

Sporadic Creutzfeldt-Jakob disease (sCJD) is a rare fatal neurodegenerative disease, and key symptoms are rapidly progressive dementia, myoclonus, ataxia, and visual disturbances. Seizures, however, are an uncommon feature in this disease [1]. We report a patient with a suspected refractory status epilepticus in the intensive care unit (ICU), who was ultimately diagnosed with sCJD. The value of the available diagnostic tests will be discussed, and we raise the question whether a combination of diagnostic tests may be sufficient for diagnosing sCJD.

## 2. Case Presentation

The case involved a 53-year-old woman. Except for a postnatal depression and a uterus extirpation, there were no previous other illnesses. About one month before admission to the ICU, she had started to exhibit aberrant emotional behavior. Seventeen days prior to ICU admission she presented with symptoms of derealization, gait abnormalities, and visual hallucinations at the neurology clinic of a local rural hospital and was admitted subsequently. Computed tomography (CT) and magnetic resonance imaging (MRI) scans were initially interpreted as normal; however, reexamination of the images at a later stage showed abnormalities similar to those found on more recent imaging. As her condition deteriorated with increasing confusion, psychosis, and a suspected catatonic state, she was transferred to the psychiatric department of our hospital 5 days later. A neurologist was consulted, and a lumbar puncture was performed. Subsequent cerebral spinal fluid (CSF) analysis showed no signs of infection and no local immunoglobulin G (IgG) production, and no neurologic diagnosis was made at that time. However, during admission, minor myoclonus of the shoulder muscles was observed, as was further impairment of consciousness. As the psychiatrist suspected a possible epileptic disorder, a neurologist was re-consulted, who decided to admit the patient to the neurology department, which was exactly one week after admission to the psychiatric ward.

**Figure 1:**
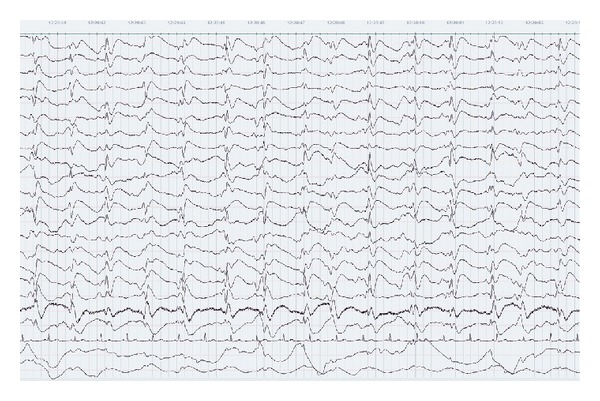
Continuous EEG registration, showing typical semiperiodic sharp-wave complexes with intervals of about 1 second.

**Figure 2:**
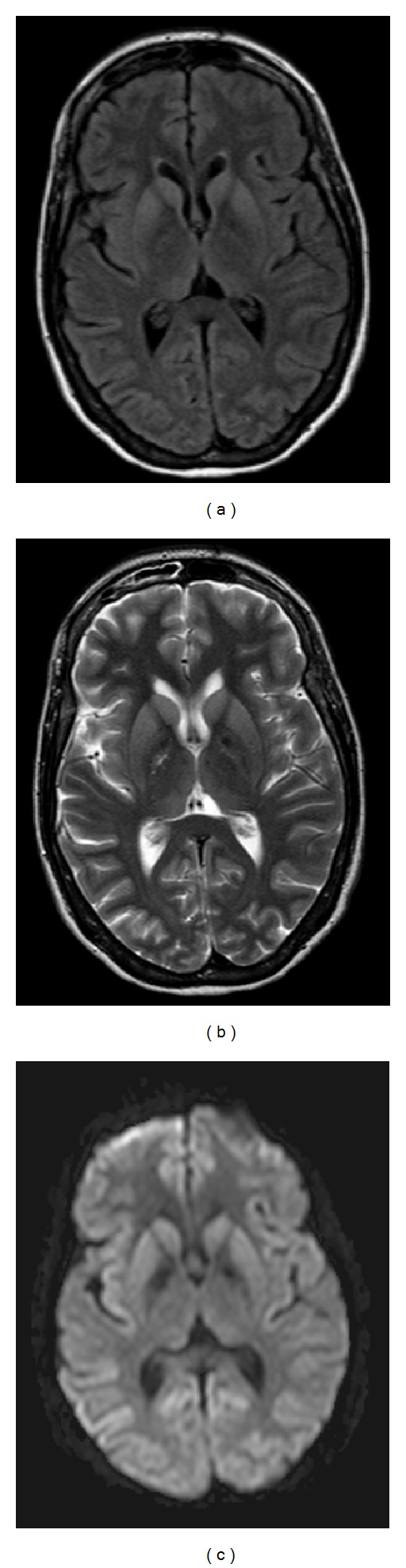
MRI findings (axial FLAIR, T2, and DWI). Subtle symmetrical hyperintensities in the caudate nucleus and putamen and less evident in the pulvinar nuclei and cerebral cortex are noticeable.The figure is constructed by J. Dorgelo.

Extensive diagnostic procedures were applied. Electroencephalography (EEG) showed a diffuse slowing of the background pattern with periods of high voltage periodic and semiperiodic sharp-wave complexes (PSWCs). Intravenous clonazepam transiently attenuated the periodic complexes. Further treatment with clonazepam, lorazepam, and valproic acid was started, and continuous EEG (cEEG) recording was applied to monitor the therapeutic effect. Unfortunately, the EEG abnormalities resolved for only a brief period, and the following days the PSWCs reappeared with intervals of about 1 second (Figure 1). A new MRI showed symmetrical hyperintensities in the caudate nucleus and putamen, and less evident in the cerebral cortex and pulvinar nuclei on T2, fluid attenuated inversion recovery (FLAIR), and diffusion weighted imaging (DWI) images (Figure 2). New CSF analysis still revealed no abnormalities. Nonetheless, a variety of possible neurotrophic infectious agents, including Borrelia, Coxiella, Whipple, Syphilis, Bartonella, Mycoplasma, and Herpes Simplex-, Varicella Zoster-, Measles-, Entero-, and Parechoviruses were investigated, which were all negative. CSF samples were sent to a university hospital laboratory for anti-N-methyl-D-aspartate (NMDA) receptor, anti-Hu, antivoltage-gated potassium (VGKC), antiglutamic acid decarboxylase (GAD) antibody, and 14-3-3 protein analysis (tau protein was not determined). A general toxicological screening was performed, which revealed no substance abuse and no plasma heavy metals. Vitamin levels (B6, B12) were normal. Thyroid disease was ruled out and autoimmune serology (antinuclear antibodies (ANA), antidouble-stranded DNA (dsDNA), antiextractable nuclear antigen (ENA), antithyroid peroxidase (TPO), lupus anticoagulant (LAC), and anticardiolipins (CL)) was negative. Urine analysis was negative for porphyrins.

In the meantime her condition worsened, and pragmatic treatment with intravenous immunoglobulins and steroids for suspected anti-NMDA receptor encephalitis were administered, however, without any improvement. On the third day after transfer from the psychiatric ward, the myoclonus of both shoulders reappeared, this time accompanied by dyskinesia of the left arm. Phenytoin treatment was started; however, her condition rapidly deteriorated as she was now nonresponsive, had a persistent downward gaze, and a deviation of the head to the right. Pathological reflexes were absent. Probably due to the extensive antiepileptic treatment the patient started to suffer from respiratory insufficiency and was admitted to the ICU, which was 5 days after admission to the neurology ward. Midazolam infusion was administered to obtain a burst-suppression pattern. Despite antiepileptic drugs being in therapeutic ranges and addition of levetiracetam, the burst-suppression pattern was difficult to realize. Therefore, a thiopental coma was induced. 

Finally, the remaining laboratory results were reported. Earlier requested CSF analysis revealed no anti-NMDA receptor antibodies, and the other paraneoplastic/autoimmune antibodies were also absent. However, CSF 14-3-3 protein was found to be positive. Based on this finding and the combination of typical EEG and MRI abnormalities, it was concluded that the patient was suffering from sCJD. It was decided to terminate all treatments, and the patient died 25 days after admission to the ICU. Autopsy confirmed the diagnosis of sCJD.

## 3. Discussion

sCJD is a rare and fatal disease, and even though several diagnostic options are available, none of these is conclusive. Therefore, the question arises: can clinicians actually rely on these diagnostic measures? This question especially concerns the process of making decisions about the continuation or termination of (supportive) treatment in the ICU.

In prion diseases CSF cell counts are usually normal. An occasional mild pleocytosis, mildly elevated protein, and oligoclonal IgG bands may be found [2]. The most common known markers for sCJD are the 14-3-3 and tau proteins. Presence of 14-3-3 proteins, which are cytosolic polypeptides, in the CSF indicates general neuronal damage, and therefore one cannot rely entirely on the presence or absence of this protein. A study by Zerr et al. has demonstrated a sensitivity of 94% and a specificity of 84% for 14-3-3 protein in sCJD [3]. It is important to use 14-3-3 determination in the appropriate clinical context, as in that case it does prove to be a highly sensitive and specific test [4]. Interestingly, negative tests with a first and positive with a second lumbar puncture have been reported [5]. Furthermore, a case report has shown a normalization of a positive initial 14-3-3 test in a patient with autopsy proven CJD, who was treated with sedatives to suppress epileptiform activity [6]. Hamlin et al. have recently reported that determination of tau protein might be preferable to 14-3-3, as its specificity was found to be significantly higher (67% versus 40%). The sensitivity found was slightly lower than that of 14-3-3 (90% versus 87%) [7]. Other researchers have also reported a higher sensitivity of tau protein (95.2% versus 76.2%) in the early diagnostics of CJD [8]. More studies need to be conducted to prove the possible superiority of tau to 14-3-3 protein in diagnosing sCJD, which also appreciate the appropriate clinical context. 

The EEG of patients with suspected sCJD often resembles a (pseudo)rhythmic pattern, thereby making it hard to distinguish the EEG from a (non)convulsive status epilepticus [9]. Typical EEG findings in sCJD comprise periodic or semiperiodic sharp-wave complexes, which consist of bi- and/or triphasic sharp-waves or mixed spikes, polyspikes, and slower waves recurring every 0.5–2 seconds [1, 10]. These PSWCs may be lateralized but frequently evolve into generalized periodic discharges (GPDs). As cEEG is used with increasing frequency in the ICU, GPDs are more often detected and have been found to be associated with a variety of diseases with diffuse grey matter involvement, including anoxia, CNS infection (Herpes Simplex encephalitis), autoimmune encephalopathy, autoimmune thyroiditis, hepatic and renal disease, drugs/toxins, metabolic encephalopathy, nonconvulsive status epilepticus, and resolution of status epilepticus [11]. The appearance of PSWCs occurs in about two-thirds of patients with sCJD; however, these PSWCs may be absent or nonspecific in the early stages [1, 10]. Variable sensitivity and specificity of the detection of these PSWCs have been reported, with 44%–66% and 74%–92%, respectively [3, 12]. PSWCs can be temporarily attenuated by sleep, auditory or painful stimuli, and intravenous administration of antiepileptic drugs (AED) but without any beneficial effects on the clinical state [1, 10]. 

MRI abnormalities found in patients with sCJD are hyperintensities in parts of the thalamus (“hockey stick sign”), basal ganglia, and cerebral cortex (“cortical ribbon sign”). FLAIR and DWI are sensitive (91%) and specific (95%) imaging techniques in detecting these abnormalities [13], even in the early phase of the disease and with absent EEG abnormalities [14, 15]. Addition of MRI to the diagnostic criteria of sCJD according to the World Health Organization (clinical features with a typical EEG and a positive 14-3-3 CSF test [16]) has proven to increase the sensitivity (98% versus 92%), however, with an equal specificity (71%) [12].

Performing a brain biopsy in patients suspected for CJD has been found useful [17]. However, because of the invasive nature, the risk of infection, and the possibility of false positive test results, brain biopsy is a last resort in the diagnostics of CJD and is only considered, when possible reversible diseases are suspected or when other diagnostic tests fail or are not feasible [18].

Unfortunately there are no pathognomonic signs for diagnosing sCJD. Therefore, one has to rely on the clinical manifestations in combination with other diagnostic tests. We believe that the differential diagnosis needs to be carefully evaluated in order not to miss a possible reversible condition [19]. However, this may lead to the application of unnecessary diagnostic procedures, such as those performed in the abovementioned case. As explained above, the sensitivity and specificity of the combination of MRI, EEG, and CSF 14-3-3 and/or tau analysis in the diagnostics of sCJD are high, with a prominent role of MRI. Although one should be aware of the abovementioned limitations of the individual tests, we propose that, when this diagnostic triad is positive, it provides sufficient grounds to justify the termination of treatment.
